# English Speakers Can Infer Pokémon Types Based on Sound Symbolism

**DOI:** 10.3389/fpsyg.2021.648948

**Published:** 2021-07-02

**Authors:** Shigeto Kawahara, Mahayana C. Godoy, Gakuji Kumagai

**Affiliations:** ^1^The Institute of Cultural and Linguistic Studies, Keio University, Minato, Japan; ^2^Center for the Humanities, Languages and Arts, Federal University of Rio Grande Do Norte, Natal, Brazil; ^3^Department of English Language and Literature, Faculty of Letters, Kansai University, Suita, Japan

**Keywords:** sound symbolism, sibilants, voiced obstruents, [p], English speakers, Pokémon types

## Abstract

Sound symbolism, systematic associations between sounds and meanings, is receiving increasing attention in linguistics, psychology and related disciplines. One general question that is currently explored in this research is what sorts of semantic properties can be symbolically represented. Against this background, within the general research paradigm which explores the nature of sound symbolism using Pokémon names, several recent studies have shown that Japanese speakers associate certain classes of sounds with notions that are as complex as Pokémon types. Specifically, Japanese speakers associate (1) sibilants with the flying type, (2) voiced obstruents with the dark type, and (3) labial consonants with the fairy type. These sound symbolic effects arguably have their roots in the phonetic properties of the sounds at issue, and hence are not expected to be specific to Japanese. The current study thus addressed the question whether these sound symbolic associations hold with native speakers of English. Two experiments show that these sound symbolic patterns were very robustly observed when the stimuli were presented in pairs; when the stimuli were presented in isolation, the effects were also tangible, although not as robust. We conclude that English speakers can associate certain types of sounds with particular Pokémon types, with an important caveat that we observed a clear task effect. Overall the current results lend some credibility to the hypothesis that those attributes that play a role in Pokémons' survival are actively signaled by sound symbolism.

## 1. Introduction

### 1.1. Theoretical Background

One of the most influential dictums that governed modern linguistic theories in the twentieth century was the thesis of arbitrariness—the relationships between sounds and meanings are essentially arbitrary (Locke, 1689; Saussure, 1916/1972; Hockett, 1959). An increasing number of studies, however, have shown that there are many cases of systematic relationships between sounds and meanings observed in human languages, and as such the thesis of arbitrariness was too strong. Such sound-meaning associations are now actively studied under the rubric of *sound symbolism*, which is a topic of extensive exploration in linguistics, psychology, cognitive science, and other related disciplines (see Nuckolls, 1999; Perniss et al., 2010; Imai and Kita, 2014; Schmidtke et al., 2014; Akita, 2015; Dingemanse et al., 2015; Lockwood and Dingemanse, 2015; Svantesson, 2017; Sidhu and Pexman, 2018; Kawahara, 2020a; Nielsen and Dingemanse, 2020 for recent reviews).

There are various reasons why sound symbolism is now considered to be an important topic of exploration. A growing body of research has shown, for example, that sound symbolism may guide first and second language acquisition to a non-trivial degree (Nygaard et al., 2009; Imai and Kita, 2014; Asano et al., 2015; Nielsen and Dingemanse, 2020). Some scholars argue that it may have played an essential role in the origin and development of human languages (Cabrera, 2012; Perniss and Vigiliocco, 2014; Perlman and Lupyan, 2018), while others claim that these sound-meaning connections may be a specific instance of more general synesthetic cross-modal perception, in which sensation in one modality can evoke sensation in another modality (Ramachandran and Hubbard, 2001; Spence, 2011; Cuskley and Kirby, 2013; Bankieris and Simner, 2015). Sound symbolism did not used to be a major topic of exploration in linguistics; however, for the reasons briefly outlined here, it has started receiving intensive attention in linguistics, psychology and neighboring fields (see Nielsen and Dingemanse, 2020 for some quantitative evidence for this research trend).

On the one hand, languages are systems which can connect sounds and meanings in an arbitrary fashion; otherwise, we would expect all the languages to use the same/similar words to express the same meanings (Locke, 1689; Saussure, 1916/1972), and that languages would not have the immense expressive powers that they do (Lupyan and Winter, 2018). At the same time, however, we are witnessing the accumulating body of evidence suggesting that speakers of various languages can systematically associate certain meanings with certain types of sounds. These studies have established, in our opinion, that *whether* sound-meaning connections are arbitrary or systematic is no longer the right question to ask—instead, the question that should be addressed is *how* arbitrariness and sound symbolism can coexist in the human language systems (Dingemanse et al., 2015); then an ensuing question is what kinds of semantic properties can be signaled via sound symbolism.

Two well-known semantic dimensions that are involved in sound symbolic associations are size and shape, which have been shown to hold across different languages (Bremner et al., 2013; Styles and Gawne, 2017; Sidhu and Pexman, 2018); for example, [a] is often judged to be larger than [i] (Sapir, 1929) by speakers of different languages (Shinohara and Kawahara, 2016), and voiceless obstruents tend to be associated with angular shapes, whereas sonorants tend to be associated with round shapes (Köhler, 1947; Ramachandran and Hubbard, 2001). There are other semantic properties which have been shown to be signaled via sound symbolism, including color, brightness, taste, weight, strength, etc. (Jakobson, 1978; Lockwood and Dingemanse, 2015; Westbury et al., 2018; Winter et al., 2019; Kawahara and Kumagai, 2021, among others), but it remains to be explored precisely what kinds of semantic concepts can be signaled via sound symbolism in natural languages, and relatedly, how complex such concepts can be (Lupyan and Winter, 2018; Westbury et al., 2018; Sidhu and Pexman, 2019).

Within this ever-growing body of studies on sound symbolism, one emerging research strategy is to explore the sound symbolic nature of natural languages using Pokémon names (Kawahara et al., 2018), a research paradigm that is now dubbed “Pokémonastics” (Shih et al., 2019). As discussed in detail by Shih et al. (2019), this approach to sound symbolism has several research advantages^1^. First, since there are many Pokémon characters (ca. 900) which all have quantitative attributes such as weight and height, it allows researchers to conduct a quantitative assessment of sound symbolism using real words^2^. Second, in natural languages, different languages assign names to a different set of real world attributes; for example, Japanese lexically distinguishes rice plant (= *ine*), cooked rice (= *gohan*), and generic rice (= *kome*), a tripartite distinction that is absent in English. Japanese, on the other hand, does not distinguish between, for example, *crying* and *moaning*. This sort of cross-linguistic difference makes it difficult to compare the sound symbolic patterns in existing words in different languages (although it is not impossible: see e.g., Wichmann et al., 2010; Pitcher et al., 2013; Blasi et al., 2016; Johansson et al., 2020 for illustrative cases of such studies). On the other hand, in the Pokémon world, the set of denotations is fixed across all languages, thereby making the cross-linguistic comparison easier. The third advantage of the Pokémonastics research is that each Pokémon character has various attributes, such as weight, height, evolution levels, strengths and types. This feature allows researchers to explore what sorts of information can be expressed via sound symbolism (Kawahara and Kumagai, 2021).

Within the framework of Pokémonastics research, this paper focuses on Pokémon types with the hope that it will (albeit modestly) contribute to the general issue addressed in the sound symbolism research discussed above. In the Pokémon game series, players collect fictional creatures called Pokémon, train them, and have them fight with other Pokémon characters. Pokémon characters are classified into several types, including, but not limited to, normal, fire, fairy, water, dragon, ghost, ground, grass, etc. Certain types of characters have (dis)advantages over other types during their battles; for example, water-type has advantages over fire-type.

Hosokawa et al. (2018) was the first study which examined whether Pokémon types are symbolically expressed in the Japanese Pokémon names. They found that labial consonants, such as [p] and [m], are overrepresented in the names of the fairy type Pokémons, whereas voiced obstruents, such as [d] and [z], are overrepresented in the villainous types (see also Uno et al., 2020). Kawahara and Kumagai (2019b) confirmed the productivity of these associations by an experimental study with Japanese speakers using nonce words. Extending on these two studies, Kawahara et al. (2020) further found that Japanese speakers associate the flying type with names containing voiceless sibilants, including [s] and [ɕ] (=voiceless alveo-palatal fricative). As discussed in further detail below, these connections are arguably grounded in the phonetic properties of these sounds, and as such they are not expected to be specific to Japanese. The current experiments therefore aim to test the cross-linguistic robustness of these sound symbolic connections with native speakers of English (see also Godoy et al., 2021 for a similar attempt with native speakers of Brazilian Portuguese).

As discussed above, the Pokémonastics research can potentially provide a useful resource for cross-linguistic comparisons of sound symbolism in natural languages. While Japanese is actively studied via experimentation within the Pokémonastics paradigm (e.g., Kawahara and Kumagai, 2019a,b, 2021; Kumagai and Kawahara, 2019; Kawahara, 2020b; Kawahara et al., 2020), we are yet to gather more data from other languages in order to more thoroughly address the cross-linguistic similarities and differences in sound symbolism. A few studies have gathered experimental data from native speakers of English and Brazilian Portuguese regarding sound symbolism signaling a pre- vs. post-evolution distinction, where post-evolution characters are generally larger, heavier and stronger (Godoy et al., 2020; Kawahara and Breiss, 2021; Kawahara and Moore, 2021). However, other than these, experimental studies on languages other than Japanese are limited. It is thus hoped that the current experiments further contribute to expanding the Pokémonastics database, which should be useful for general sound symbolism research (cf. Shih et al., 2019).

### 1.2. The Three Sound Symbolic Connections Tested

The three sound symbolic connections that were tested in this study are: (1) sibilants = flying, (2) voiced obstruents = dark, and (3) [p] (as a representative of labial consonants) = fairy. In this subsection we expand on each of these sound symbolic associations.

#### 1.2.1. Sibilants = Flying

The investigation of the first sound symbolic association, sibilants = flying, was inspired by the remarks of two ancient writers. First, Socrates suggested that [s] and [z] are suited for words that represent wind and vibration (in Classical Greek), because the production of these sounds accompanies strong breath (Cratylus: 427). Second, the Upanishads suggested that sibilants represent air and sky. To reinterpret these remarks from the perspective of modern phonetics, sibilants (including [s] and [ʃ] in English) involve a large amount of oral airflow during their production (Mielke, 2011), and this aspect of these sounds may be iconically mapped onto the image of wind, and, by extension, flying (see also Paraise et al., 2014 for the iconic relationship between high frequency sounds—of which sibilants are typical examples—and the notion of elevation).

Kawahara et al. (2020) presented Japanese speakers with pairs of nonce words in which one member contained sibilants and the other did not (e.g., [saɾoɕɕuu] vs. [taɾokkuu]), and asked them to judge which member of the pairs was better suited for the flying type Pokémon. Their results suggest that Japanese speakers associate nonce names containing sibilants with the flying type above the chance level. One aim of the current study is to examine whether English speakers make the same sound symbolic association.

#### 1.2.2. Voiced Obstruents = Dark

The second association was first identified as an existing sound symbolic pattern in the Japanese Pokémon lexicon by Hosokawa et al. (2018). Prior to their studies, it was already known that Japanese monster names and villainous characters' names frequently contain voiced obstruents (= [b], [d], [ɡ] and [z]) (Kawahara, 2017; Kawahara and Monou, 2017). Building on these observations, Hosokawa et al. (2018) showed that voiced obstruents are overrepresented in villainous Pokémon characters, where they defined “villainous” as consisting of dark, ghost and poison types.

In general, voiced obstruents are associated with negative images in Japanese (Suzuki, 1962; Hamano, 1998; Kubozono, 1999; Kawahara, 2017), and arguably this sound symbolic connection may have its roots in the articulatory difficulty of producing voiced obstruents (Ohala, 1983). In order to maintain vocal fold vibration, the air pressure level has to be lower in the oral cavity than in the subglottal cavity. However, intraoral air pressure is raised when the airflow needed for vocal fold vibration becomes trapped in the oral cavity due to the obstruent closure/constriction. This results in difficulty maintaining vocal fold vibration, and speakers need to resort to various articulatory adjustments to expand their oral cavity (Ohala, 1983; Westbury and Keating, 1986; Proctor et al., 2010). Because of this articulatory challenge, many languages phonologically avoid voiced obstruents in favor of voiceless obstruents (Hayes, 1999; Hayes and Steriade, 2004). It would not be too surprising if this articulatory challenge is projected onto general negative images (Kawahara, 2017; Uno et al., 2020).

In fact, this association between voiced obstruents and negativity manifests itself in English, as well as in Japanese. Shinohara and Kawahara (2009) presented pairs of pictures of the same object, one in its clean state and the other in its dirty soiled state (e.g., a clean sponge vs. a dirty soiled sponge). Along with these pictures, they presented nonce words containing voiced obstruents and those containing voiceless obstruents (e.g., [zabe] vs. [sape]). Their results showed that both Japanese and English speakers tend to associate nonce words containing voiced obstruents with pictures of dirty soiled items. Another relevant observation is the finding that in the set of Disney characters names in English, villains' names are more likely to contain voiced obstruents than non-villains' names (Hosokawa et al., 2018; Uno et al., 2020).

Building on these observations, the current study tests whether English speakers associate voiced obstruents with villainous characters in the Pokémon world, taking the dark type as a representative of villains. We used dark type as the representative, because the dark type literally means the “evil” type (=*aku*) in the original Pokémon series in Japanese. It is explained as such in the instructions of the experiments reported below.

#### 1.2.3. [p] = Fairy

The third hypothesis, like the second hypothesis, was also first identified by Hosokawa et al. (2018) as one of the statistically reliable tendencies in the Japanese Pokémon names. The general observation that lies behind the hypothesis was that labial consonants—those that are produced by using lips, including [p] and [m]—are generally associated with the image of babies, as evidenced by the fact, for example, that labial consonants are overrepresented in baby diaper names in Japanese, both in the set of existing names and in the new names elicited via experimentation (Kumagai and Kawahara, 2020). Labial consonants are also shown to be overrepresented in the names of PreCure girls—a TV series that is popular among young girls in Japan—who are cute fighters (Kawahara, 2019). Along the same line, Hosokawa et al. (2018) show that bilabial consonants are overrepresented in the fairy type Pokémon characters, which tend to be, like babies and PreCure girls, cute. This association found by Hosokawa et al. (2018) was shown to be productive by a follow-up nonce-word experiment (Kawahara and Kumagai, 2019b): given a pair of non-existing names like [paɾapiɾu] and [kaɾakiɾu], Japanese speakers tend to choose the former for fairy type characters.

This sound symbolic association is hypothesized to arise from the observation that labial consonants appear frequently in early speech and babbling (e.g., Jakobson, 1941; MacNeilage et al., 1997; Ota, 2015). The current study thus addresses the question whether, like Japanese speakers, English speakers also associate labial consonants with cute, fairy characters^3^. The current study used [p] as a representative of labials, because it is the consonant that has been judged to be outstandingly cute (Kumagai, 2019). Whether English speakers associate other labial consonants with fairy type characters is yet to be explored in future experimentation. Testing [b] would be particularly interesting, because of its ambivalent nature (Uno et al., 2020): its labiality would be suited for fairy names, whereas its aspect as a voiced obstruent may not be (cf. Kawahara and Kumagai, 2019b who found that Japanese speakers do judge [b] to be suitable for fairy type Pokémons).

## 2. Experiment 1

To recap, the current experiment tested three sound symbolic associations that have been shown to hold for Japanese speakers: (1) sibilants = flying, (2) voiced obstruents = dark, and (3) [p] = fairy. In addition to testing these patterns, we also examined a task effect by conducting two experiments: in Experiment 1, the stimuli were presented in isolation, whereas in Experiment 2, the stimuli were presented in pairs.

Many experiments on sound symbolism present the stimuli in pairs (see Westbury et al., 2018 for a very comprehensive overview). For instance, Sapir (1929), one of the classic experimental studies on sound symbolism, presented two nonce words (*mal* vs. *mil*) and asked the participants which one means “a big table” and which one means “a small table.” In establishing the *bouba-kiki* effect, Ramachandran and Hubbard (2001) presented the two stimuli (*bouba* and *kiki*) as a pair, and asked which one corresponds to a round figure and which one corresponds to an angular figure. The same holds for Köhler (1947) who used *takete* vs. *maluma*. The two previous experimental studies on Pokémon types that the current study significantly builds upon (Kawahara and Kumagai, 2019b; Kawahara et al., 2020) also deploy this format.

This format, more generally known as a 2AFC (2 Alternative Forced Choice) task—has been the common practice in sound symbolic research, but this raises the question of how robustly sound symbolic patterns hold when the stimuli are presented in isolation (again, see Westbury et al., 2018). Generally speaking, the task would be easier for the participants if the stimuli are presented in pairs than in isolation^4^. Since many of the previous studies in Pokémonastics—as well as other studies in sound symbolism—use a 2AFC format, we took advantage of this opportunity to examine whether sound symbolic associations under question hold even when the stimuli are presented in isolation.

### 2.1. Methods

#### 2.1.1. The Stimuli

The list of stimuli used in this experiment is shown in Table 1. For all the pairs, the target consonants appeared twice within each stimulus. The vowels and other target consonants were controlled between the two conditions.

**Table 1:** The list of stimuli used in Experiment 1.

**(a) Names with sibilants**	**(b) Control**
Silshin	Tiltin
Salshim	Taltim
Sulshur	Tulkur
Shieshen	Kieten
Shilsun	Kiltun
Shalshick	Kaltick
Shelshim	Kelkim
**(c) Names with voiced obstruents**	**(d) Control**
Bringlin	Prinklin
Branzlam	Pranslam
Drinzlin	Trinslin
Dramblum	Tramplum
Grimblin	Krimplin
Grenzlin	Krenslin
Zegdum	Sektum
Zumgul	Sumkul
**(e) Names with [p]**	**(f) Control**
Peepol	Teetol
Polpen	Tolken
Pafpil	Tastil
Pimpock	Tintock
Paapair	Kaakair
Pupmir	Kukmir
Pepmil	Kekmil

For the sibilant condition, the target words contained two sibilants. There were three items that started with [s] and four items that started with [ʃ] (“sh”), but most of them had [ʃ] internally, because word-internal orthographic ‘s' in English can often be pronounced as [z]. We focused on voiceless sibilants in this study because voiced sibilants can be produced as approximants, as the intraoral air pressure cannot be raised too much to maintain vocal fold vibration (Ohala, 1983). The control condition had three items that started with [t] and four items that started with [k]. While the stimulus items were not directly paired in Experiment 1, [s] was matched with [t] and [ʃ] was matched with [k], because articulatorily speaking, [t] and [s] are front consonants, whereas [ʃ] and [k] are back consonants (Mann and Repp, 1981).

For the voiced obstruent condition, the target items began with either [b], [d], [ɡ] or [z] (two items each), and contained one or more word-internal voiced obstruents. The control condition consisted of words that contained corresponding voiceless obstruents with the same manner and place of articulation. For the last condition, the target words started with [p] and contained an additional word-internal [p]. The control consisted of words that contain either [t] or [k]^5^.

Since Pokémon names are often communicated in written form, and since the previous Pokémonastics experiments used orthographic stimuli, the current experiment followed that methodology (Kawahara and Kumagai, 2019a; Kawahara and Moore, 2021). Yet, an experiment with auditory stimuli may be warranted in future studies given the possible influences of orthography on sound symbolism (Cuskley et al., 2017). We note, however, Sidhu et al. (2016) have demonstrated that sound symbolism holds beyond the influences of orthography. With this caveat in mind, the participants were nevertheless asked to read each name silently in their head before making their decision.

#### 2.1.2. Procedure

The experiment was administered online using SurveyMonkey. The first page of the experiment was a consent form, which was approved by the first author's institute. The second page presented our qualification questions, and only those who fulfilled all four of the following conditions were allowed to proceed: (1) they are a native speaker of English, (2) they are familiar with Pokémon, (3) they are not already familiar with sound symbolism, (4) and they have not participated in a Pokémonastics experiment before.

**Figure 1:**
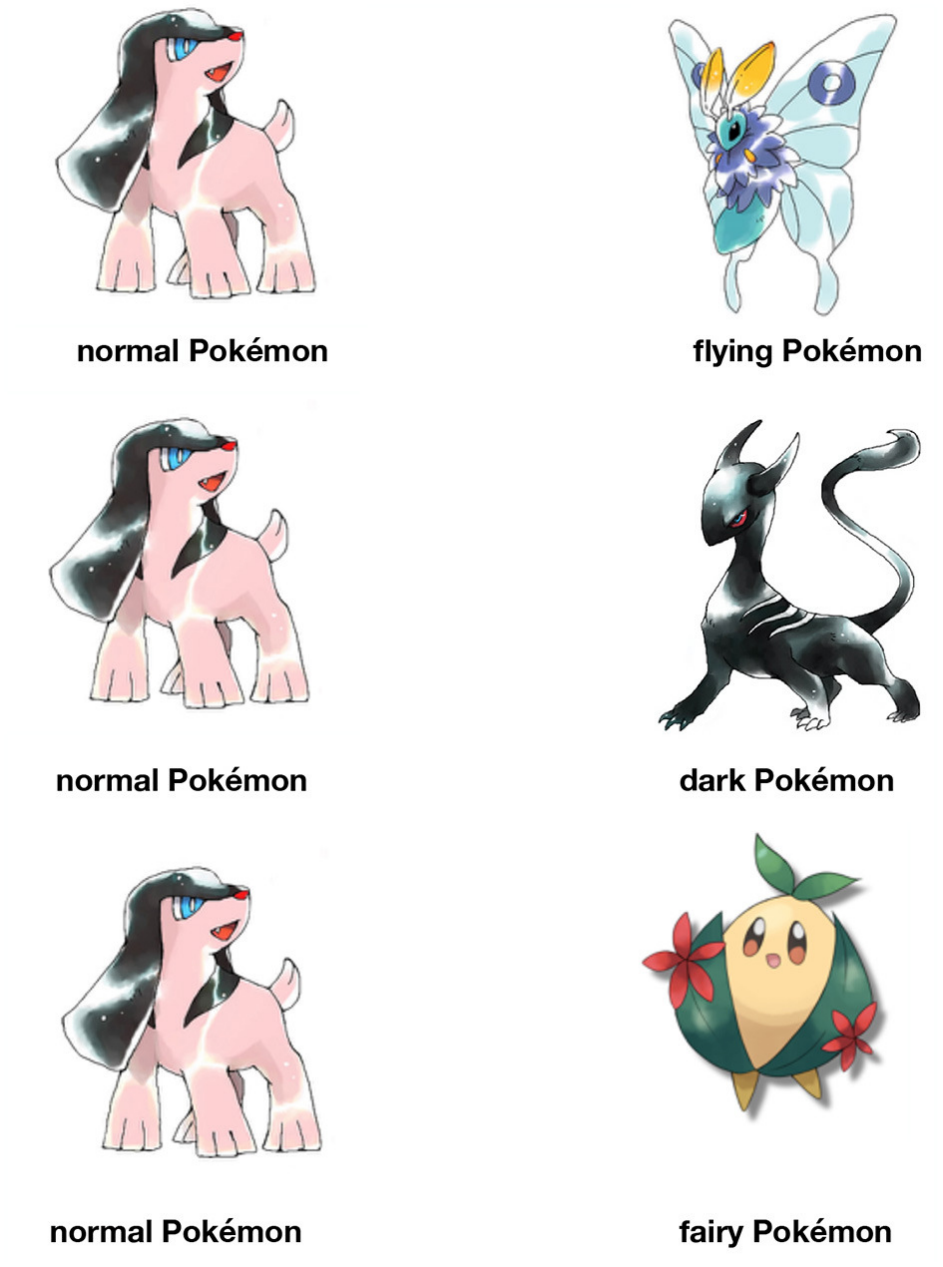
Pictures used to illustrate each of the three types of Pokémon in the current experiment. These are non-existing Pokémon characters originally drawn by a digital artist *toto-mame* (https://t0t0mo.jimdofree.com). They are used in the experiment with permission from the artist.

The entire experiment was blocked into three sections, each of which tested one sound symbolic effect on type, in the order of flying type, dark type, and fairy type. The first page within each section introduced a difference between one type of Pokémon, which was contrasted with a normal type of Pokémon, using a pair of pictures shown in Figure 1. The participants were asked to answer whether they understood the difference between the two types. The flying type was defined as those that fly in the sky. The dark type was defined as those that are villainous and evil. The fairy type was those that were cute.

Each name was presented in isolation, and the participants were asked to choose which type each name fits better. They were also told that there are no “right” or “wrong” answers, and were asked to provide their answer using their intuitive feelings. The order of the stimuli within each block was randomized per participant.

After the main trials, the participants were asked to report how familiar they are with Pokémon using a 7 point scale, where 1 is labeled “Never touched it,” 4 is labeled “so so,” and 7 is labeled “Pokémon is my life.”

#### 2.1.3. The Participants

The responses were collected using the buy response function of SurveyMonkey. A total of 159 English speakers participated in the experiment. Eleven of them were excluded based on the exclusion criteria listed in §2.1.2. Thirteen participants were excluded because they responded that one or more differences in type was not clear. The data from the remaining 135 participants were analyzed. Among them, 56 of them were male, with one not reporting their gender.

#### 2.1.4. Analysis

To statistically analyze the data, we fit a Bayesian mixed effects logistic regression model. There are various advantages of using Bayesian analyses instead of a more traditional frequentist approach; for accessible introduction to Bayesian analyses in psychology and linguistic research, see e.g., Nicemboim and Vasishth (2016), Kruschke and Liddell (2018), and Franke and Roettger (2019); Kruschke (2014) is a thorough and accessible introductory book on this general statistical approach. A slightly more technical illustration as well as application of Bayesian analyses in linguistic/phonetic studies using the brms package (Bürkner, 2017), also used in the current study, can be found in Vasishth et al. (2018).

Bayesian analyses take into account both prior knowledge (if any) and the data at hand to yield a range of posterior estimates for parameter values that are of interest. In logistic regression analyses, we are primarily interested in the estimate of the slope coefficient (*β*_1_) of a particular effect; i.e., for the case at hand, the slope coefficient of the sound symbolic effect.

One particular advantage of Bayesian analyses is that we can interpret the posterior distributions of *β*-coefficients as directly reflecting the degrees of our belief—or (un-)certainty—about the estimates of the parameter that we are interested in. One common heuristic to interpret these posterior distributions, which is roughly analogous to significance testing in a frequentist approach, is to examine its 95% Credible Intervals (CIs) of the distributions, which can be obtained by discarding the extreme 2.5% of the posterior samples at the upper and lower ends. If 95% CI does not include 0, we can be reasonably confident that the effect meaningfully impacts the responses, or put differently, *β*_1_ at issue is not equivalent to 0.

However, one important advantage of Bayesian analyses is that we can move beyond the “significant vs. non-significant” dichotomy that is usually embraced in a frequentist analysis (see e.g., Kruschke, 2014; Nicemboim and Vasishth, 2016; Vasishth et al., 2018; Franke and Roettger, 2019). Instead, we can, for example, calculate the proportion of posterior values that are larger than a particular value. To be more specific, in order to examine whether a particular sound increases certain responses, we can analyze the whole posterior distribution of its *β*_1_-coefficient, and calculate the proportion of posterior values that are above 0. A more conservative approach is to examine the ROPE (Region Of Practical Equivalence) of a point hypothesis that *β*_1_ = 0 (Kruschke, 2014; Kruschke and Liddell, 2018). To do so, we take the effect size of 0.1 (Cohen, 1988) of a standardized parameter value to define the range of ROPE. In a logistic regression model, the standardized parameter value can be approximated as $\frac{\pi }{\sqrt{3}}$ (= 1.8) (Makowski et al., 2019); thus, we calculated the proportions of posterior samples that are more extreme than 0.18.

In short, we calculated the 95% CI of *β*_1_ as well as *p*(*β*_1_>0) and *p*(*β*_1_>0.18). All the posterior samples are available in the supplementary file, and interested readers are welcome to examine them in other ways—another virtue of a Bayesian approach.

The details of the actual implementation are as follows. Analyses were implemented using the brms package (Bürkner, 2017) and R (R Development Core Team, 1993). The dependent variable was whether or not the response was the target type. The predictor contained a fixed effect of a sound type, a random intercept of items, and a random slope and intercept of participants. The weakly informative priors (the default setting for brms) were used. Four chains were run. The $\hat{R}$-values were all 1.00, suggesting that the chains mixed successfully. We first ran 2,000 iterations with 1,000 warmups. When the ESS values were too large, more iterations (e.g., 4,000) were run, and the last 1,000 iterations were interpreted. See the accompanying R markdown file provided as the supplementary file for complete details.

### 2.2. Results

**Figure 2:**
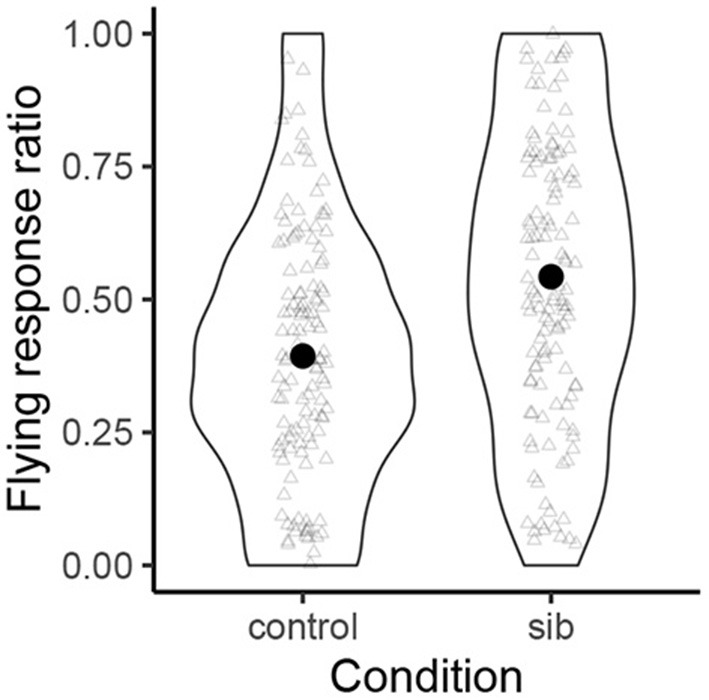
The normalized probability distribution of “flying response” ratios. The black circles represent the grand means. The transparent triangles represent each individual point (jittered).

Figure 2 shows violin plots which represent the normalized probability distributions of by-participant “flying response” ratios for those names with sibilants (right) and those names without (left). Transparent triangles represent data from each participant. The black circles within each violin plot represent the grand averages. On average, the names with sibilants were more likely to be judged to be the names of the flying type than the were control names (54.3 vs. 39.4%).

The mean of the slope coefficient (*β*_1_) for the difference between the control condition and the sibilant condition was positive (0.77). The 95% CI of *β*_1_ was [0.21, 1.35]. Since this interval does not include zero, we can be reasonably confident that names with sibilants meaningfully increase “the flying response” with respect to the control names. Examination of all the posterior samples shows that 99.6% of the posterior estimates of this slope coefficient were positive, and 98.2% of them were above 0.18. We can thus be at least 98% confident that names with sibilants increase the flying responses with respect to the control names.

**Figure 3:**
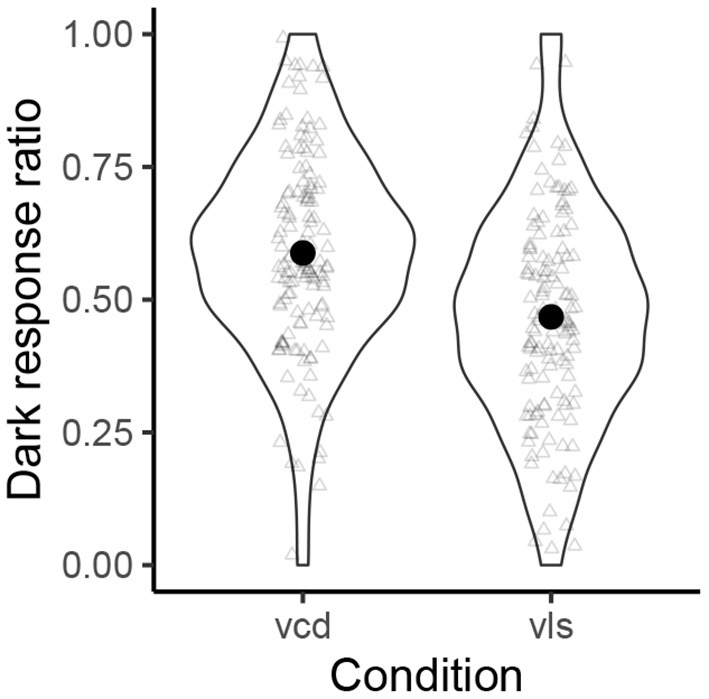
The normalized probability distributions of the “dark response” ratios.

Figure 3 shows the violin plots of the normalized probability distribution of the by-participant “dark response” ratios for those names with voiced obstruents (left) and those names with voiceless obstruents (right). Overall, names with voiced obstruents were more likely to be associated with the dark type Pokémon characters than the control names with voiceless obstruents (58.8 vs. 46.8%). Since the “voiced (vcd)” condition was the baseline and the coefficient tells how the “voiceless (vls)” condition lowered “dark” responses, the mean value of the *β*_1_ coefficient was negative (–0.55), with its 95% CI being [–1.38, 0.26]. Since we are interested in how the voiceless condition lowers dark responses, we calculated the proportions of posterior estimates that are negative and those that are lower than –0.18. The results suggest *p*(*β*_1_ < 0) = 91.3% and *p*(*β*_1_ < −0.18) = 82.3%.

**Figure 4:**
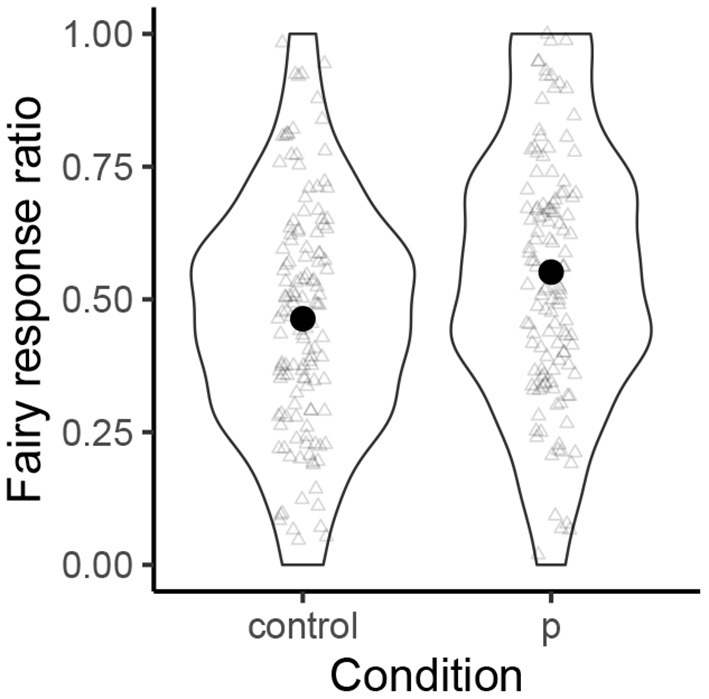
The normalized probability distributions of the “fairy response” ratios.

Figure 4 shows the results for the fairy condition. The names with [p] were more likely to be associated with the fairy type than the control names (55.1% vs. 46.3%). The mean of the *β*_1_ coefficient is 0.42, with its 95% CI being [–0.24, 1.08]. The examination of all the posterior samples of the *β*_1_ coefficient shows that 90.1% of them were positive, and 77.0% of them were above 0.18.

### 2.3. Discussion

All three conditions showed responses in the expected direction. None of the effects are deterministic; i.e., names with particular phonological categories are not categorically associated with a certain Pokémon type, although we can identify individual responses in the violin plots that were categorical. Such a stochastic nature of sound symbolism, however, is the norm rather than the exception (Dingemanse, 2018; Kawahara et al., 2019).

One question that arises from the current results is to what extent the sound symbolic effects observed in the experiments are affected by familiarity with Pokémon, albeit most previous Pokémonastic studies did not find substantial effects of familiarity on their results (e.g., Kawahara and Kumagai, 2019b; Kawahara and Moore, 2021). To address this question, we calculated an effect size for each participant by subtracting the target type response ratio given the control stimuli from the target type response ratio given the target stimuli. Since the scale of familiarity obtained in the post-experimental questionnaire was non-continuous, a non-parametric Spearman correlation was calculated between effect sizes and familiarity ratings for each of the three sound symbolic effects. The results show that no significant correlations hold between these two measures (flying: *ρ* = 0.00; dark: *ρ* = –0.08; fairy: *ρ* = 0.07). The sound symbolic effects observed in the experiments, therefore, seem to hold independently of how familiar the participants were with Pokémon.

In the current experiment, how confident we can be about the sound symbolic effects differed among the three conditions; i.e., the probability of *β*_1_ being larger/smaller than the ROPE threshold was 98.2% for the flying condition, 82.3% for the dark condition, 77.0% for the fairy condition; and the probability of *β*_1_ being in the expected direction was 99.6%, 91.3%, and 90.1%, respectively. We took advantage of the Bayesian approach and offered several numerical indices of how confident we can be about these sound symbolic effects, rather than making a dichotomous “yes significant” vs. “not significant” decision often deployed in a frequentist approach. Heuristically, it seems safe to conclude that the sibilant=flying connection seems to be a very robust sound symbolic connection. On the other hand, the [p]=fairy connection may not be too reliable, although the result still seems encouraging. The connection between dark type and voiced obstruents lies somewhere in-between.

There are two possible interpretations regarding why we did not identify a robust effect of, say, the [p]=fairy connection in the current experiment. One interpretation is to posit that English speakers do not make this sound symbolic association at all^6^. We hesitate to accept this interpretation because many of the posterior samples of *β*_1_ were in the expected direction, and even if we take the most conservative approach, more than 75% of the posterior samples were above 0.18.

An alternative possibility that we would like to explore next is that there is indeed a sound symbolic effect between [p] and the fairy type, but this effect was not very clearly observed in this experimental format. First, as stated at the beginning of this section, it is more challenging for the participants to make a judgment when stimuli are presented in isolation than in pairs—this is one crucial difference between the current experiment and Kawahara and Kumagai (2019b), who found a robust effect of labiality with Japanese speakers^7^.

Second, it is possible that since the stimuli are presented in isolation, the participants' responses were influenced by other segments that are contained in the stimuli. For example, *Polpen* was judged more likely to be the normal type than the fairy type, despite the fact that it contains two [p]s. This may be because the initial vowel [o] is the “large” vowel in English (Newman, 1933), and hence may have been judged to be inappropriate for the fairy type. Likewise, *Tintok* was judged to be the fairy type almost as frequently as the normal type, which may be because of its initial [i], which is the “small” vowel in English (Newman, 1933)^8^. In order to further explore the sound symbolic effects under question, the next experiment presented the stimuli in pairs.

## 3. Experiment 2

### 3.1. Methods

The methods for Experiment 2 were almost identical to those for Experiment 1, unless otherwise noted. Table 2 lists the stimulus pairs used in Experiment 2. Most of the stimuli were the same as those used in Experiment 1, except that the first and the third conditions contained one additional test pair. In this experiment, all the conditions had 8 pairs.

**Table 2:** The list of stimuli used in Experiment 2.

**(a) Sibilants = flying**
Silshin vs. Tiltin
Salshim vs. Taltim
Sulshur vs. Tulkur
Surshum vs. Turkum
Shieshen vs. Kieten
Shilsun vs. Kiltun
Shalshick vs. Kaltick
Shelshim vs. Kelkim
**(b) Voiced obstruents = dark**
Bringlin vs. Prinklin
Branzlam vs. Pranslam
Drinzlin vs. Trinslin
Dramblum vs. Tramplum
Grimblin vs. Krimplin
Grenzlin vs. Krenslin
Zegdum vs. Sektum
Zumgul vs. Sumkul
**(c) [p] = fairy**
Peepol vs. Teetol
Polpen vs. Tolken
Pafpil vs. Tastil
Pimpock vs. Tintock
Paapair vs. Kaakair
Pupmir vs. Kukmir
Pepmil vs. Kekmil
Parpil vs. Karkil

As in Experiment 1, the responses were collected using the buy response function in SurveyMonkey. A total of 157 native speakers of English participated in the experiment. Thirteen of them were excluded because they did not fulfill all the participation requirements (see §2.1.2). One participant did not finish the experiment. Eight were not sure about at least one of the three type differences. The data from the remaining 135 participants entered into the following analysis. Among them 66 were male. One of the exclusion criteria (“have not participated in a Pokémonastics experiment before”) ensured no overlap between the participants for Experiment 1 and those for Experiment 2.

The procedure for the experiment was identical to that of Experiment 1, except that the stimuli were presented in pairs. As in Experiment 1, the participants were asked to read the stimuli and use their auditory impression to make their responses.

To fit a mixed effects model using the results obtained in a 2AFC format, we followed the methodology proposed by Daland et al. (2011), which has advantages over other possible alternatives (see their footnote 5)—this is also the methodology often used in other Pokémonastics experiments when analyzing data obtained using a 2AFC format (Kawahara and Kumagai, 2019a,b; Kawahara et al., 2020). Specifically, one trial was split into two observations, each corresponding to one member of a stimulus pair. The other details are almost identical to those of Experiment 1, except the models did not include an item-specific random intercept, because each item contributes to both an expected response and an unexpected response. The fixed effect (“expectedness”) was coded as –0.5 vs. 0.5. See the accompanying markdown file for complete details.

### 3.2. Results

**Figure 5:**
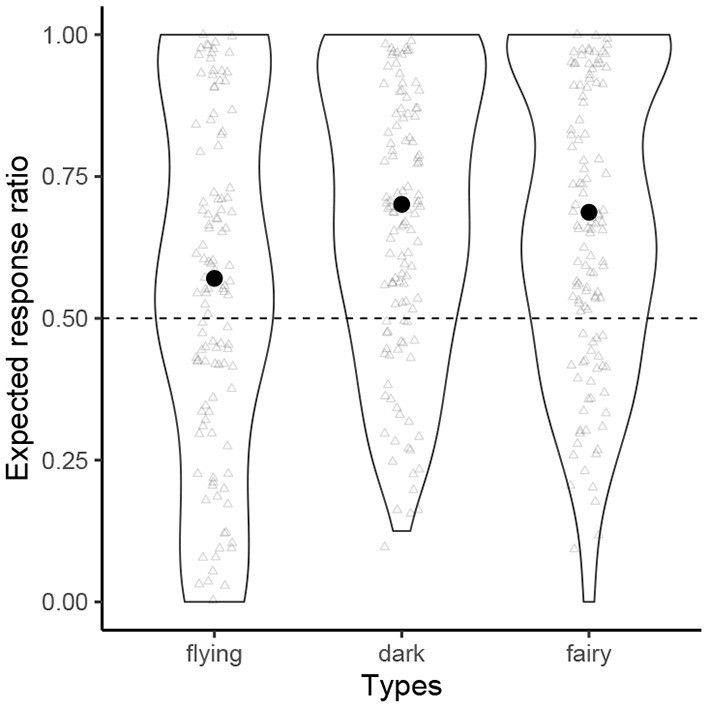
The probability distribution of “expected response” ratios for each condition.

Figure 5 shows the normalized probability distribution of the by-participant expected response ratios for each condition, where “expected” indicates (1) sibilants = flying, (2) voiced obstruents = dark, (3) [p] = fairy. The grand averages are all above the chance level (flying: 0.57; dark 0.70; fairy: 0.69), although we observe that some speakers showed responses that were below chance.

The means of the *β*_1_ coefficients were all positive (flying = 0.57, dark = 1.71, fairy = 1.58), and none of their Bayesian 95% CIs included zero (flying [0.40, 0.74]; dark [1.53, 1.89]; fairy [1.39, 1.76]), and in fact, none of the posterior samples of the *β*_1_ coefficients were lower than 0.18. For this format of the experiment, we can be 100% confident that each sound symbolic principle meaningfully affected the participants' responses.

### 3.3. Discussion

Experiment 2 has confirmed the productivity of all the three sound symbolic connections that were of interest. Taken together with the results of Experiment 1, we conclude that English speakers make sound symbolic connections between certain classes of sounds and particular types of characters in Pokémon games, just as Japanese speakers do, with an important caveat that we observed a clear task effect—the sound symbolic effects were more robustly observed in an experiment in which the stimuli were presented in pairs, i.e., in a 2AFC format.

Recall however that the previous experiments conducted with Japanese speakers deployed a 2AFC format (Kawahara and Kumagai, 2019b; Kawahara et al., 2020), just like our Experiment 2. The current results were thus no less reliable than the previous Pokémonastic studies. With this said, how sensitive Japanese speakers are to these sound symbolic associations needs to be studied using an experimental format like Experiment 1 in future work. In fact, echoing Westbury et al. (2018), more generally speaking, this future task applies to many sound symbolic patterns that have been studied using a 2AFC format.

At this point we would like to address one potential general concern about the 2AFC format that was raised by Westbury et al. (2018) in the context of the current experiment. In the 2AFC format that is deployed in Experiment 2, it might be that the control names had some sound symbolic associations with the images of the normal Pokémons, which could explain the skews in the responses observed in Experiment 2. We doubt this possibility, because the normal type of Pokémon was not associated with any particular feature, at least in this experiment. Neither do we have reasons to believe that sounds used in control names in our comparisons have particular sound symbolic values, which would be associated with the normal type.

Finally, as with Experiment 1, we examined whether the results of Experiment 2 were affected by the participants' familiarity with Pokémon, by calculating the correlation between the expected response ratio from each participant and the reported familiarity with Pokémon. The results were that no substantial correlation was found (flying: *ρ* = 0.06; dark: *ρ* = –0.09; fairy: *ρ* = 0.07). As was the case with Experiment 1, the sound symbolic patterns seem to hold regardless of how familiar the participants are with Pokémon.

## 4. Inference From the Existing Patterns?

One question that arises from the current experimental results is whether these sound symbolic patterns hold in the existing set of English Pokémon names, or whether English speakers could infer Pokémon types based on their tacit knowledge about sound symbolism in the experiments. To address this question, we examined the dataset created by Shih et al. (2019), which includes all the data for English Pokémon names up to the 7th generation (total *N* = 802)^9^.

Table 3 shows the distribution of names containing sibilants in the flying type and normal type; contrary to our experimental results, names containing sibilants were more common for the normal type than for the flying type, although this difference was not significant (χ^2^(1) = 1.22, *n*.*s*.).

**Table 3:** The distributions of names containing voiceless sibilants in the flying type and normal type in the existing English Pokémon names.

	**Flying type**	**Normal type**
Contain sibilants	19 (19%)	29 (26.4%)
Contain no sibilants	81	81
Total	100	110

Table 4 shows the distribution of names containing voiced obstruents in the dark Pokémons and normal Pokémons. It shows that voiced obstruents are slightly more overrepresented in the dark Pokémons, but this difference was not significant (χ^2^(1) = 1.29, *n*.*s*.).

**Table 4:** The distributions of names containing voiced obstruents in the dark type and normal type.

	**Dark type**	**Normal type**
Contain voiced obstruents	28 (59.6%)	53 (48.2%)
Contain no voiced obstruents	19	57
Total	47	110

Finally, Table 5 shows the distribution of names containing [p] in the fairy type and normal type, which shows that [p] is, contrary to the experimental results, more common in the normal type. This difference is not statistically significant, however (χ^2^(1) = 0.62, *n*.*s*.).

**Table 5:** The distributions of names containing [p] in the fairy type and normal type.

	**Fairy type**	**Normal type**
Contain [p]	9 (19.1%)	26 (23.6%)
Contain no [p]	38	84
Total	47	110

Overall, none of the sound symbolic effects are visible in the existing English Pokémon names. This result reveals an interesting difference between English and Japanese, as recall that Hosokawa et al. (2018) showed that two of the three sound symbolic patterns under question hold in the existing Pokémon names in Japanese. (The connection between sibilants and the flying type is not observed in the existing Japanese names: Kawahara et al., 2020.) The reason why the existing English names do not exhibit these sound symbolic connections may be because Pokémon characters were created and named in Japan first, and they were translated into English sometimes by using real words to describe those characters; for instance, *hitokage*, a small lizard-like character which blows fire, is named *Charmander*, based on *charcoal* and *salamander*. After all, for many words, sound-meaning associations are arbitrary (Saussure, 1916/1972; Hockett, 1959); therefore, together with the semantic restrictions imposed during the translation process, the English names may have ended up not being very sound symbolic^10^ (although see Shih et al., 2019 who show that some sound symbolic effects are observable in the existing English Pokémon names as well).

Nevertheless, we find it interesting that when English speakers are given nonce words with appropriate phonological properties, they are able to, albeit probabilistically, make the same sound-symbolic associations that Japanese speakers do. The overall results therefore support the thesis that arbitrariness and sound symbolic connections can co-reside within a single linguistic system, or put differently, just because existing words are arbitrary, it does not mean that speakers do not have intuitions about possible sound-symbolic connections.

## 5. Conclusion

We started with a general question regarding sound symbolic effects in natural languages: what kinds of semantic properties can be signaled via sound symbolism, and how complex can these properties be? The current experiments have shown that notions as complex as Pokémon types can be symbolically represented. We find this result to be intriguing as they show that sound symbolism is not limited to simple semantic notions such as size and shape.

We also find it encouraging that those sound symbolic associations that are tested in the experiments have plausible bases in the phonetic and/or phonological properties of the sounds at issue. To recap, sibilants involve large amounts of oral airflow during their production which is required to cause frication (Mielke, 2011), and this phonetic property may be iconically mapped onto the notion of wind, and by extension, flying. Voiced obstruents may be associated with general negative images, because of their articulatory challenge (Ohala, 1983). Labial consonants, particularly [p], may be associated with the image of cuteness, because those are the typical sounds that are produced by babies (Jakobson, 1941). It would not be surprising if such sound symbolic patterns, which are grounded in their phonetic and phonological properties, are shared across different languages. We do not intend to pretend that testing these effects in just two languages—Japanese and English—suffices to establish the universality of sound symbolism, yet the current finding offers a good start for future cross-linguistic investigations (though see also Godoy et al., 2021).

Having established that English speakers too can infer Pokémon types from sound symbolism, we would like to end this paper by briefly discussing what Shih et al. (2019) conclude based on an extensive cross-linguistic comparison of Pokémon names. In the real world, we observe various types of sound symbolic effects to signal gender differences (Sidhu and Pexman, 2019); for instance, male names are more likely to contain obstruents than female names (e.g., *Eric* vs. *Erin*: Cassidy et al., 1999; Sidhu and Pexman, 2019). On the other hand, we do not observe robust sound symbolic effects to signal gender differences in the Pokémon world. This difference between the real world and the Pokémon world arises maybe because finding a mate is important for reproduction, i.e., survival, in the real world, but not so much in the Pokémon world. This hypothesis is further supported by the fact that Pokémon strength status is sound symbolically signaled across languages, together with the fact that Pokémon characters fight with each other; i.e., Pokémon strength is important for their survival.

Thus, sound symbolism may be actively deployed to signal those attributes that are important for their survival in that world (Uno et al., 2020). Types play a non-trivial role in Pokémon battles (e.g., fairy type has advantages over dark type), and therefore, it is predicted that types constitute an attribute that should be signaled by sound symbolism. While the current study lends further support to this idea, it also raises a few new questions. One is whether types other than flying, dark, and fairy can be symbolically represented. Another is whether the sound symbolic patterns tested in the current study also hold for speakers of languages other than English and Japanese (see Godoy et al., 2021). More generally, can we observe sound symbolic effects for any properties that are relevant for survival and reproduction in the real world? These questions can and should be tested via future experimentation.

All in all, the current experiments have shown that English speakers can associate certain types of sounds with certain Pokémon types, as do also Japanese speakers. This parallel may not come as too much of a surprise, to the extent that the sound-meaning associations are grounded in the phonetic and phonological properties of the sounds at issue. Finally, the fact that the sound symbolic associations are not observed in the existing English Pokémon names but yet can be identified by English participants with nonce words shows that arbitrariness and sound symbolism can co-exist within a single linguistic system.

## Data Availability Statement

The datasets presented in this study can be found in online repositories. The names of the repository/repositories and accession number(s) can be found below: https://osf.io/2m34s/.

## Ethics Statement

The studies involving human participants were reviewed and approved by The Keio Institute of Cultural and Linguistic Studies. The participants read the consent form before participating in this study.

## Author Contributions

SK analyzed the results and wrote the initial version of the manuscript. MG and GK revised the manuscript. All authors contributed to the design and execution of the experiment as well as the discussion of the results.

## Conflict of Interest

The authors declare that the research was conducted in the absence of any commercial or financial relationships that could be construed as a potential conflict of interest.

## Footnotes

^1^However, Pokémonastics, which analyzes made-up names of fictional characters, is not meant to replace the studies of sound symbolism using real words; it is instead meant to complement other related studies on sound symbolism. See Kawahara and Breiss (2021) for some extended discussion on this point.

^2^A reviewer raised an interesting challenge related to this thesis—the set of denotations that needs to be expressed by a language in the real world is much larger than this *N*, and it would be reasonable to conjecture that the expressive power that is required in such situations may not allow sound symbolism to persist as strongly, at least compared to the Pokémon universe, given that the number of phonemic contrasts is limited (cf. Lupyan and Winter, 2018). The general prediction of this conjecture is that the larger the set of denotations that needs to be expressed, the less sound symbolic the words should become. It is beyond the scope of the current paper to address this prediction, but it can be empirically tested by way of artificial language creation experiments, for example. If we ask participants to create names for a set of new objects (cf. Perlman and Lupyan, 2018), the larger the set of new objects that needs to be named, the less sound symbolic the created names are predicted to be.

^3^A previous Pokémonastics experiment has shown that given pairs of nonce words containing labial consonants and those containing coronal consonants (e.g., *Meepen* vs. *Neeten*), English speakers tend to choose the former for pre-evolution characters more often than for post-evolution characters (Kawahara and Moore, 2021).

^4^In fact, in Signal Detection Theory, a quantitive psychophysical measure of sensitivity (“d-prime”) is adjusted by $\sqrt{2}$ when the stimuli are presented in pairs in a 2AFC format (Macmillan and Creelman, 2005).

^5^The fact that the first and third hypotheses had 7×2 items whereas the second hypothesis had 8×2 items is due to the fact that SurveyMonkey maximally allows 50 questions in order for us to use the buy response function (see §2.1.3 below). It was necessary to include the consent form, the qualification questions, and illustrations of each type, which made it impossible to have 8×2 items for all three hypotheses.

^6^The Bayesian approach we took suggests that this is at best too strong a conclusion. In order for us to accept *β*_1_=0, the 95% CI of *β*_1_ should be contained in the ROPE of that point estimate, which is [–0.18, 0.18] (Kruschke, 2014; Kruschke and Liddell, 2018; Makowski et al., 2019).

^7^Another potential factor which may have contributed to the difference between English and Japanese can be that the concept of “fairy” —and also that of “cuteness” —may differ between English speakers and Japanese speakers. As Donna Erickson pointed out (p.c.), “sprite” may have been a better term than “a fairy”, because (English) fairies usually have wings. It might thus be interesting to run a similar follow-up experiment using the word “sprite” to describe that type of Pokémons.

^8^This is truly a *post-hoc* speculation, but this name may have sounded too much like *Tinker Bell* or *Tink*. Since this is a *post-hoc* hypothesis, we will not re-run the statistics excluding this item to avoid p-hacking (Simmons et al., 2011).

^9^We are grateful to Stephanie Shih and her colleagues for letting us use the database. Due to the data sharing agreement, this dataset cannot be publicly made available.

^10^A reviewer suggested an interesting alternative explanation regarding how this difference between Japanese and English may have arisen: suppose that English speakers have an impression that a particular set of sounds are associated with “Japanese-sounding” names, then such associations could have masked the sound symbolic patterns in the existing Pokémon names, assuming that English Pokémon names were created to sound like Japanese names. This hypothesis is plausible given the previous psycholinguistic finding that English speakers can detect “foreignness” in some sounds (e.g., [ʒ] is associated with “French-ness”: Gelbart, 2005). This hypothesis can be more explicitly tested by exploring specifically which sounds English speakers associate with “Japanese-ness”.
